# Antitumoral and Anti-inflammatory Roles of Somatostatin and Its Analogs in Hepatocellular Carcinoma

**DOI:** 10.1155/2021/1840069

**Published:** 2021-11-27

**Authors:** Argyrios Periferakis, Georgios Tsigas, Aristodemos-Theodoros Periferakis, Ioana Anca Badarau, Andreea-Elena Scheau, Mircea Tampa, Simona Roxana Georgescu, Andreea Cristiana Didilescu, Cristian Scheau, Constantin Caruntu

**Affiliations:** ^1^Department of Physiology, The “Carol Davila” University of Medicine and Pharmacy, Bucharest 050474, Romania; ^2^Department of Radiology and Medical Imaging, Fundeni Clinical Institute, Bucharest 022328, Romania; ^3^Department of Dermatology, The “Carol Davila” University of Medicine and Pharmacy, Bucharest 020021, Romania; ^4^Department of Dermatology, Victor Babes Clinical Hospital for Infectious Diseases, Bucharest 030303, Romania; ^5^Division of Embryology, Faculty of Dental Medicine, The “Carol Davila” University of Medicine and Pharmacy, Bucharest 050474, Romania; ^6^”Prof. N.C. Paulescu” National Institute of Diabetes, Nutrition and Metabolic Diseases, Bucharest 011233, Romania

## Abstract

Hepatocellular carcinoma (HCC) is the most common primary liver cancer and affects about 8% of cirrhotic patients, with a recurrence rate of over 50%. There are numerous therapies available for the treatment of HCC, depending on cancer staging and condition of the patient. The complexity of the treatment is also justified by the unique pathogenesis of HCC that involves intricate processes such as chronic inflammation, fibrosis, and multiple molecular carcinogenesis events. During the last three decades, multiple in vivo and in vitro experiments have used somatostatin and its analogs (SSAs) to reduce the proliferative and metastatic potential of hepatoma cells by inducing their apoptosis and reducing angiogenesis and the inflammatory component of HCC. Most experiments have proven successful, revealing several different pathways and mechanisms corresponding to the aforementioned functions. Moreover, a correlation between specific effects and expression of somatostatin receptors (SSTRs) was observed in the studied cells. Clinical trials have tested either somatostatin or an analog, alone or in combination with other drugs, to explore the potential effects on HCC patients, in various stages of the disease. While the majority of these clinical trials exhibited minor to moderate success, some other studies were inconclusive or even reported negative outcomes. A complete evaluation of the efficacy of somatostatin and SSAs is still the matter of intense debate, and, if deemed useful, these substances may play a beneficial role in the management of HCC patients.

## 1. Introduction

Liver cancer is the fourth most frequent type of cancer and has been a rising cause of concern for the global medical community [1]. Despite significant diagnostic and therapeutical advances, hepatocellular carcinoma (HCC) still has recurrence rates of over 50%, even after aggressive treatments, such as curative resection [2–4]. Recent studies show that the majority of patients are at risk of recurrence, and this is attributable to intrahepatic metastasis or multicentric hepatocarcinogenesis [5].

HCC, the most frequent primary liver cancer, is more common in males and certain ethnic groups [6]. Its incidence increases markedly among patients with chronic viral hepatic infections and cirrhosis. Up to 8% of cirrhotic patients might develop HCC at some point in their lives [1]. Despite these viral causes, chronic alcohol consumption remains the leading cause of hepatocellular carcinoma [7], and this is in part explained by the fact that alcoholism is by far more prevalent than hepatitis B and C infections [8]. The risk of HCC is also increased by metabolic conditions such as diabetes mellitus and obesity. This etiological variety implies that patients with HCC will also carry the burden of the underlying disease, and their management will require a more complex approach, often involving an interdisciplinary team to address cardiovascular, neurological, metabolic, or renal complications as well as any other associated pathologies [9–11]. Also, the presence of rare subtypes of HCC or atypical presentations can further delay the treatment and worsen patient prognosis [12, 13].

There are several therapies employed against HCC, ranging from potentially curative to palliative and symptomatic [14]. The most promising treatments are those involving liver resection, transplantation, and local ablation [15], but for a host of causes, only about 20% of the HCC patients can benefit from such radical treatments [16, 17]. Consequently, most HCC patients will be subjected to palliative or symptomatic treatment, with survivability of about 40%, at most [18]. Treatment options are evaluated based on the patient's condition and the severity of the carcinoma itself, among other factors [19]. While there are various evaluation systems employed, the BCLC staging system is the most widely adopted in Europe and the USA and is of interest to all medical specialties involved in the management of HCC patients [14, 20].

Due to the limited therapeutic options, especially in advanced HCC, and the relatively low efficiency of the available drugs in terms of improving overall survival, there is a high need for the development of new treatments [21]. A large number of new molecular targeted drugs are tested in clinical trials [22]. Recently, several alternative treatments for HCC have come into focus. The use of oncolytic viruses, the application of stem cell research and peptide vaccines, and even the modulation of the intestinal microbiota have been considered [23]. The use of natural compounds is increasingly investigated and also seems promising, especially when used in association with conventional treatment [24–27].

Another important therapeutic solution for HCC may be the use of somatostatin and its analogs (SSAs), which have been employed mostly in patients towards the later stages of HCC. In this review, we summarize the published experimental and clinical results and present the current research on the correlation between the action of somatostatin and SSAs in HCC and the expression of somatostatin receptors (SSTRs).

## 2. Somatostatin and Hepatocellular Carcinoma

### 2.1. General Actions of Somatostatin

Somatostatin can be thought of as a wide-ranging inhibitory peptide, with diverse functions depending on the target tissue [28]. More specifically, it may function as a neurotransmitter, a neuromodulator, an endocrine hormone, or a paracrine factor, while its role as a trophic factor has also been proposed [29, 30]. Physiologically, its levels are very low due to its prompt degradation by ubiquitous peptidases [31]. It exists in two isoforms known as SRIF14 and SRIF28—SRIF standing for somatotropin release inhibitory peptide. It is noteworthy that the inhibitory effects of somatostatin are directed not only towards the release of peptides per se but also towards the target tissues; it is possible for somatostatin to simultaneously and independently block the release of gastrin and of gastric secretion [32]. Frequently, the inhibitory effects of somatostatin are so potent as to be able to inhibit the associated peptides regardless of the type and intensity of the stimuli [28].

Somatostatin was isolated from the gastrointestinal tract, genitourinary system, heart, eyes, thyroid, thymus, and skin [33–35]. The presence of somatostatin was also identified in the central nervous system, where the concentration of somatostatin is sufficient to inhibit growth hormone release from the pituitary gland via the hypothalamic-pituitary axis [36].

### 2.2. Somatostatin in the Treatment of Hepatocellular Carcinoma

The importance of neuroendocrine factors in various cancers has been emphasized in recent research [30, 37, 38]. Studies on somatostatin, ever since its discovery, have demonstrated its inhibitory effects on the secretion of numerous hormones, as mentioned above. So, it is reasonable to assume that its administration by reducing glucagon, gastrin, and insulin levels will have an adverse effect on the cells targeted by these polypeptides acting as trophic factors [39, 40]. This might exert at least an antiproliferative if not an apoptotic effect.

It is known that one of the major antiproliferative actions of somatostatin is exerted via the mitogen-activated protein kinase (MAPK) pathway, which leads to cell cycle arrest in the G1 phase [41]. This has been shown to occur due to the upregulation of the p21cip1/waf1 and p27kip1 kinase inhibitors [42]. Nevertheless, there are other signaling mechanisms associated with this function, as we will further elaborate in this paper. The specific mechanism depends upon the type of receptor expressed in the studied cells [43]. Somatostatin can induce apoptosis, either by following a pathway involving the p53 protein [44] or by other p53-independent mechanisms [45, 46]. It can also exert the same effects by increasing the expression of the Fas-Fas ligand system, which promotes apoptosis [47].

The antineoplastic effect of somatostatin may be attributed to its potential to modulate immune pathways, although further research is required on the topic [48]. It is also possible that somatostatin reduces oxidative stress [49] and NO production [50], which further contribute to the antineoplastic effect.

Somatostatin also reduces the levels of proinflammatory cytokines in rat liver stellate cells [51], and this anti-inflammatory action may be beneficial in HCC. Other effects on stellate cells have also been reported, but this remains subject to further research [52, 53]. Furthermore, somatostatin may reduce the activity of MMPs which are associated with Kupffer cells [43].

The mentioned antitumoral effects of somatostatin on HCC are presented in Table 1.

The current research consensus for the HCC metastasis seems to indicate that the PEBP1 (RKIP) gene is the main culprit behind the invasive behavior. In general, the expression of PEBP1 is found to be much lower in cancer cells [54], and therefore, its upregulation might limit the metastatic potential. PEBP1 can act by inhibiting metastasis, acting as a tumor suppressor gene [55]. Huang et al. showed that increased concentration of somatostatin in SK-Hep-1 and HepG2 cancer cell lines can be correlated with increased expression of the PEBP1 gene and therefore with decreased invasive and metastatic potential [56].

Apart from influencing the endogenous expression of specific proteins which regulate the invasive and metastatic potential of the HCC cells, somatostatin may also be used for the direct and indirect downregulation of proteins related to the destruction of the extracellular matrix, the prerequisite for cancer invasion and metastasis. Highly invasive cancers such as HCC are characterized by abnormal activity levels of both intracellular and extracellular molecules. A paradigm of intracellular proteins was that of the RKIP as mentioned before, while extracellular related molecule research focuses on matrix metalloproteinases (MMPs) [56].

Recent data has shown MMPs as promising targets to avert or minimize the invasiveness of cancer cells, while the overexpression of these enzymes presents a direct correlation with cancer metastasis prognosis [57, 58]. In the pathogenesis of HCC, MMPs are involved in many processes related directly or indirectly to pathogenesis, such as fibrosis, weakening of the matrix, and tissue destruction [59–61]. While antibodies against these specific enzymes are currently experimented with, their use in integrated clinical practice remains elusive [62]. Somatostatin may regulate the activity of MMPs directly or indirectly, via IGF [63].

Therefore, somatostatin exhibits two types of antimetastatic potential effects: the direct control on antimetastatic genes and the influence on the activity of specific enzymes that promote metastasis. These functions of somatostatin are also exerted by its analogs, and there were several reports of metastasis halt or even reversal [64, 65]. However, no definitive evaluations for this effect of either somatostatin or SSAs are available at the moment, and further research is required on the subject.

### 2.3. Somatostatin Analogs in the Treatment of Hepatocellular Carcinoma

In modern clinical practice, somatostatin has been replaced, in many instances, by analogs due to the easier administration and the potentially severe side effects of using pure somatostatin [66]. More specifically, somatostatin administration must be performed intravenously, due to its half-life of approximately 3 min; therefore, its window of action is rather limited. Moreover, postinfusion hypersecretion rebound is also present, and growth hormone and insulin are secreted in pathological values, shortly after the activity of the administered somatostatin has ended [67].

There are several somatostatin-derived synthetic analogs available for the treatment of HCC. Early research has shown that somatostatin analogs inhibit tumor growth in animals [68, 69]. However, a consensus on the efficacy of such a treatment has not been reached, mainly due to the different research parameters which make comparison and evaluation of results difficult [1].

While there is little difference in the affinity of somatostatin receptors for SRIF-14 and SRIF-28, there is a marked affinity difference in the binding of the somatostatin analogs. All somatostatin analogs have a very high affinity for SST_1_ and generally higher affinity than somatostatin for the other SSTRs. Whereas octreotide and lanreotide have a great affinity for SST_2_ and SST_5_ and to a lesser extent SST_3_ [70], pasireotide has a high affinity for receptors SST_1_, SST_3_, and SST_5_ [71]. Since different types of SSTs have been identified in different types of HCC, it may be of paramount importance to identify the type of SST or SSTs which are more prevalent and adjust the SSA treatment accordingly [52, 72–75].

The numerous experiments on the action of somatostatin and its analogs (e.g., [44, 52, 76–85]) did not always exhibit a unified corpus of conclusions. While many researchers replicated the originally determined antiproliferative and/or apoptotic effects, others did not manage that, and some even had conflicting results, depending on the dose of somatostatin or SSA employed.

However, it is the general research consensus that both somatostatin and its analogs exert a direct antiproliferative and apoptotic effect, which is modulated by the different types of somatostatin receptors [43]. The different mechanisms associated with these actions seem to be SSTR-dependent. While some SSTRs may be implicated in the same type of responses, they are associated with different signaling pathways, as seen in Table 2.

Similar to somatostatin, SSAs can induce cell cycle arrest by stopping the hepatoma cells in the G_1_ phase, through specific receptors and the corresponding signaling pathways [86, 87, 93, 95]. The activation of SSTR_2_ and/or its heterodimerization with SSTR_3_ can induce apoptosis [42, 94]. The activation of these receptors yields the same results, whether it is performed by somatostatin or by SSAs. Inhibitory effects of octreotide and other analogs on liver tumors have been reported [96–98]. The inhibition of trophic factor secretion by somatostatin and its analogs might act as an antiproliferative and an apoptotic factor on HCC [39, 40].

Somatostatin analogs also activate the Fas-Fas ligand system [47], which induces the formation of the death-inducing signaling complex and forms an integral part of the anticancer immune function [99]. The ligation of Fas with FasL results in the activation of caspase-mediated apoptosis [100–102]. However, such mechanisms depend upon the serum levels of the analog. For the specific case of the octreotide, in vitro studies have revealed a concentration threshold, which if exceeded, the octreotide exerts antiproliferative effects, but if not, it actually promotes proliferation [43, 96].

Recent data showed that opioids bind to the somatostatin receptors, inducing the PTP signaling cascade [103]. The opioid growth factor and its receptor are also capable of halting cancer cell proliferation by inhibiting DNA replication [104]. This is especially important since functional opioid receptors have not been identified in HCC cell lines [43].

Octreotide has also shown direct and indirect inhibitory effects on angiogenesis [105–107]. Direct inhibition involves the SSTRs while indirect effects occur through inhibition of the vascular endothelial growth factor or of the adenylyl cyclase [39, 108, 109]. An octreotide and celecoxib combination has been successfully employed as an antiangiogenic agent [110].

It is possible that the SSA-induced immune pathway modulation may exert an antineoplastic effect, but this needs to be corroborated by further research [48, 111, 112].

Octreotide reduces the inflammatory component of HCC through a dual effect of decreasing the concentration of proinflammatory cytokines while increasing the anti-inflammatory cytokines [113]. It was also suggested that liver macrophages are downregulated by octreotide [114]. In addition, the TGFb1 secretion by the Kupffer cells is inhibited when these cells are exposed to octreotide, and this may contribute to the anti-inflammatory effects of SSAs [114]. SSAs also demonstrate antineoplastic effects by reducing oxidative stress and NO production [49, 50, 115].

### 2.4. In Vitro and Animal Experiments Involving Somatostatin Analogs

The initial success of somatostatin analogs in in vitro experiments in the treatment of other types of carcinomas [93] led to the investigation of the potential of this compound on patients suffering from HCC [77]. Initial reports showed that octreotide exerts dose-dependent apoptotic effects on Bel-7402 hepatoma cells, therefore introducing it as a potential antineoplastic drug [80]. Further research used the longer-lasting lanreotide, in a series of in vitro and in vivo experiments; the in vitro data showed lanreotide to exert a dose-dependent apoptotic potential on human HepG2 cells in the S-phase [78]. Wang et al. [79] determined that octreotide induces apoptosis and dose-dependent inhibition of cell proliferation on SMMC-7721 HCC cells. They also recorded tumor growth inhibition when xenografting the cell line to mice which was considered a consequence of an octreotide-induced decrease in DNA synthesis.

The study of Liu et al. [44], using octreotide on both normal liver cells and HCC cells, verified the apoptotic effect on the pathological cell lines and correlated it with the expression of SSTR_3_, which is uniquely expressed in those cells. Again, the effect was dose-dependent. In most of these studies, a decrease in the synthesis of *α*-fetoprotein, a marker for proliferative activity, was observed. However, Reynaert et al. [52] did not manage to replicate the antiproliferative effect of somatostatin analogs, when using specific SSTR agonists, but noted that the metastatic potential of the cells was significantly reduced and correlated with the expression of SSTR_1_.

Hua et al. [83] did not succeed in reproducing the results of previous studies [44, 77, 78] on the Bel-7402 cell line and noted no quantifiable apoptotic effect but noticed that after exposure to octreotide, the SSTR_2_ expression levels were decreased. On the other hand, when these cells were xenografted to rats, it prevented the growth of the xenograft and HCC development, similar to previous reports [52].

An interesting experiment was performed by Xie et al. [82] who used two cell lines, HepG2 and HepG2x, the latter having a transfected HBV X gene. This gene codes for a small peptide, of 154 amino acids, which stimulates several cellular transduction pathways, in many cell types, including hepatocytes [116]. They noted that the apoptosis of the first cell line was significantly increased, but the second cell line was unresponsive, even when octreotide was used in combination with lamivudine, an antiretroviral medication, frequently used to treat AIDS/HIV and chronic hepatitis B. This was positively correlated with the decreased expression of SSTR_2_ and SSTR_5_ in the cells transfected with the HBV X gene.

Grant et al. [88] used a HEK 293 cell clone which expressed both a hemagglutinin- (HA-) tagged SSTR_2_ and a c-Myc-tagged SSTR_5_. This cell clone was specifically chosen for its relatively low SSTR expression levels, compared to physiological conditions [117]. They determined that the p21 and p27Kip1 cyclin-dependent inhibitors, associated with SSTR_2_, were involved in cell cycle arrest. Ma et al. used the SMMC-7221 cell line and found that apoptosis positively correlates with the dosage and exposure time to octreotide and is achieved through the activation of the Fas-FasL ligand system [47]. Tsagarakis et al. reported that high doses of octreotide inhibited proliferation, while low doses of octreotide promoted proliferation in the HepG2 line [96].

Klironomos et al. used hepatic stellate cells isolated from rats, to study the effects of somatostatin in their proliferation in correlation with the expression of SSTRs [85]. They determined that the effects of octreotide were subject to the cytokine microenvironment of those cells, but they noted that collagen production was reduced, mirroring the results of the research of Reynaert et al. who used somatostatin on the same cell line [118].

By using octreotide, lanreotide, and SOM230, another somatostatin analog, Lü et al. replicated the apoptotic effect of earlier researches using the Bel-7402 cell line [84]. Their study had also an in vivo component where the tumor cell lines were xenografted on mice, and it was observed that survival and quality of life were improved. These effects were attributed to the variations of SSTR expression in the cancer cells.

Octreotide also inhibited tumor progress in rats after partial hepatectomy [119], and further experiments confirmed these findings [120, 121]. The combination of a COX 2 inhibitor with SSAs demonstrated antiproliferative effects [98], a combination also proving successful in rabbits [97]. Following transcatheter arterial embolization, a combination treatment of octreotide with celecoxib also inhibited metastasis and angiogenesis [122]. A recent experiment on Sprague-Dawley rats has also raised the possibility of using octreotide preventatively in nonalcoholic steatosis, to prevent HCC development [123].

Lanreotide was also proven to have antiproliferative and apoptotic actions [124] and also to decrease fibrosis and angiogenesis in a series of animal experiments [125, 126]. In addition, lanreotide administration in rats was proven to prevent malignant transformation, an effect associated, most probably, with the reduction of oxidative stress [115]. The results of the mentioned in vitro experiments with SSAs are summarized in Table 3.

### 2.5. Clinical Evidence Involving Somatostatin Analogs as Single Treatment

An early clinical trial on patients with HCC using subcutaneous octreotide reported an improved median survival rate, compared to the control group; also, there were reports of tumor size decrease, and in some patients, the tumor disappeared [77]. The study concluded that octreotide administration can improve life expectancy and the quality of inoperable patients.

Positive results of octreotide were also reported in a retrospective study although the small size of the studied sample does not allow for a statistical evaluation [127].

Several case reports recorded excellent results when using octreotide for the treatment of advanced HCC [64, 128]; lanreotide was also found effective in metastatic HCC, and the positive response was correlated with SSTR_2_ expression [65].

In the clinical trial of Raderer et al., intramuscular injection of lanreotide was used in patients with inoperable HCC and resulted in partial response to treatment and improvement in the quality of life for some patients [78]. The consensus of the researchers was that, most likely, the doses of lanreotide administered were suboptimal and that higher doses might achieve more significant results. However, further clinical trials using octreotide demonstrated an overall increase in survivability [129–136].

Conversely, there were clinical trials that reported no significant difference between the control and the treated group [137–140]. The use of pasireotide as a second-line treatment was mostly unsuccessful in another trial [141]. However, possible explanations for these results include an improper choice of the control and the treatment groups and the trial parameters. Furthermore, there were retrospective observational studies that found octreotide administration to be ineffective in altering the survival rate of the patients, albeit 40% of them were alcoholics, which is a mitigating factor in the potential success of such therapies [103].

A study on less than 30 patients tested octreotide and found limited beneficial clinical results [142]. Although the results may seem disheartening, as pointed out by Samonakis et al., the choice of patients and the statistical processing of results may leave a lot to be desired [143]. The study of Cebon et al. [144], which used octreotide, mentioned that patients reported improvement in some symptoms, but it did not record any overall amelioration in the quality of life of the patients examined, while some also minor anticancer activity of the octreotide was registered. Overall, it was believed, however, that this trial had not been a success. Another factor that evaluates the results of this study was the variable length of the treatment, which was subject to disease progression and/or toxicity of the compound or withdrawal at the patient's or the doctor's discretion. In addition, 22% of the patients were alcoholics, a factor that must be considered in the objective assessment of the trials' results. The study of Dimitroulopoulos et al. [145] reported the doubling of the survival rate of patients with hepatitis-induced cirrhosis, who expressed SSTRs. Patients with a lack of SSTR expression did not respond to the treatment.

A small observational study using octreotide by Shah et al. [146] had some mixed results, with 6 patients, out of the original 22, surviving past 10 months, with advanced HCC developed on hepatitis B infection, and being of Asian descent, potentially indicating a racial aspect of the response to therapy.

### 2.6. Clinical Evidence Involving Somatostatin Analogs in Combined Treatment

Following the encouraging results of using SSAs in the treatment of HCC, further clinical trials were developed combining SSAs with other forms of treatment.

In one trial, octreotide was combined with tamoxifen but failed to provide any clear benefit, when compared to the results of the control group, which was treated solely with tamoxifen [147]. Pan et al. used tamoxifen, combining it with octreotide and chemotherapy, and demonstrated positive results in about 40% of the patients whose treatment included octreotide [81]. About 52% of the participants of this trial were alcoholics. Octreotide was also combined with sorafenib, and moderately positive results were achieved, but further evaluation of the potential of this pharmacological combination is required [1, 148].

Octreotide was also tested in combination with rofecoxib in the randomized trial of Treiber et al. [149]. Positive results were associated with the IGF and VEGF levels of the patients, indicating the potential antiangiogenetic effect of the applied combination. When octreotide was combined with heparin, in posttranscatheter arterial chemoembolization (TACE) patients, during the yearly follow-up, the metastasis incidence decreased in the treated patients; the control group had been treated with heparin only [150].

Everolimus and pasireotide were also tested in combination, but no clear benefit could be discerned and a quarter of the treated patients developed hyperglycemia [151]. About 60% of the patients were alcoholics, and treatment discontinuation was brought about by disease progression. Finally, lanreotide and celecoxib were combined with quite positive results; serum VEGF levels were correlated with positive response [152]. The combination of octreotide with sorafenib seemed to reduce serum NO levels, and this is possible evidence of a reduction of oxidative stress, thus signifying a potential antineoplastic effect [153].

Moreover, SSAs were also considered supplementary therapeutic means to surgical interventions in several clinical trials. Montella et al. used octreotide following radiofrequency ablation, with positive results, in inoperable patients [154]. Additionally, Liu et al. tested the effects of octreotide administration, following curative surgery on HCC hepatitis B-positive patients, and determined that survivability was higher in those patients whose HCC cells had high SSTR expression [155]. However, neither of these two studies were randomized nor had a control group; therefore, supplementary data is required for a more accurate assessment of their results. The trials mentioned above are summarized in Table 4.

It should be noted that a number of studies reporting negative results for the use of SSAs were conducted on patients belonging to the most advanced stage of the disease (i.e., BCLC stage D), where survival is very limited and the treatment is usually symptomatic, not contributing to the improvement of life expectancy. Therefore, it appears that SSAs are more suitable for HCC patients belonging to BCLC stages where kinase inhibitors, monoclonal antibodies, immune checkpoint inhibitors, or other new agents are employed, an opinion also shared by other authors [43].

## 3. Conclusions

Both somatostatin and its analogs have proved useful, in a clinical setting, for the treatment of various malignancies, and their use in oncologic therapy is still a subject of research. From both in vivo and in vitro experiments, it has been determined that both somatostatin and SSAs share apoptotic, antiproliferative, antineoplastic, and anti-inflammatory properties, although some of these effects can be exerted via different mechanisms. A distinct difference is that the antiangiogenic effect has so far been associated only with SSAs.

The results of the clinical trials involving SSAs are mixed, mostly due to the heterogeneity of the trials in regard to design, lot selection, and inclusion criteria. However, the positive reports are encouraging. There seems to be potential use for SSAs, especially considering that many patients may be ineligible for chemotherapy. SSAs may also provide a viable alternative to other emerging therapies for HCC with minor or major side effects where the gravity depends mostly on the specifics of the patient.

Overall, somatostatin and its analogs appear to be promising candidates in the treatment of HCC, and further studies regarding their effectiveness and safety may reveal their definitive role in the multimodal approach of HCC patients.

## Figures and Tables

**Table 1:** Classification of therapeutic actions of somatostatin on HCC.

Level	Effect type	Mechanism	Reference
Cellular	Antiproliferative	MAP kinase pathway—G1 phase arrest	[41, 42]
		Other mechanisms (specific receptors)	[43]
		Trophic factor secretion inhibition	[39, 40]
	Apoptotic	p53-dependent	[44]
		p53-independent	[45, 46]
		Trophic factor secretion inhibition	[39, 40]
		Fas-Fas ligand expression increase	[47]
Systemic	Antineoplastic	Immune pathway modulation	[48]
		Reduction of oxidative stress	[49]
		Reduction of NO production	[50]
	Anti-inflammatory	Decrease of proinflammatory cytokine levels	[51]
		Potential direct effect on stellate cells	[52, 53]
		Reduction of Kupffer cell-related MMP activity	[43]

**Table 2:** Specific effects of SSTR stimulation and associated signaling pathways [66, 86–93].

Receptor	Strongest agonist	Signaling pathway	Effect	Reference
SSTR_1_	All SSAs	Tyrosine phosphatase SHP-2 stimulation Induction of MAPK-ERK pathway and p21:Waf1:Cip1 Adenylyl cyclase modulation	Cell cycle arrest Reduced metastatic potential	[41, 52]
SSTR_2_	Vapreotide	Modulation of the ERK_1/2_ pathway and activation of SHP-1, SHP-2, and PTP*η* Modulation of the p21 and p27kip1 pathways Adenylyl cyclase modulation	Apoptosis Cell cycle arrest; antineoplastic	[82, 83, 87, 88]
SSTR_3_	Lanreotide	Adenylyl cyclase modulation	Apoptosis	[42, 94]
SSTR_4_	Octreotide	MAP kinase pathway, Ca^2+^-channels, K^+^-channels, and Na^+^-H+ antiporter Adenylyl cyclase modulation	Cell cycle arrest	[41]
SSTR_5_	Octreotide	Guanylate cyclase inhibition and MAP kinase-ERK pathway Adenylyl cyclase modulation	Cell cycle arrest Antineoplastic Apoptosis	[41, 82, 84]

**Table 3:** In vitro experiments with SSAs and their results.

Somatostatin analog	Cell line	Mechanism	Result	References
SSA RC-160+CCK	Chinese Hamster Ovary (CHO) cells	Inhibition of CCK-induced intracellular cGMP formation and activation of p42-MAP kinase phosphorylation and activity	Inhibition of cell proliferation in response to the administration of cholecystokinin	[93]
Lanreotide	Human HepG2 cells	Potential action through the SST3 and/or insulin and IGF	Antiproliferative effect proportional to the SSA dose established	[78]
Octreotide	Human BEL-7402 cells	Potentially correlated to the antineoplastic effect of SSA	Antiproliferative effect proportional to the SSA dose established	[80]
Octreotide	SMMC-7721 HCC cells	Antineoplastic action potentially attributable to decreased DNA synthesis	Antiproliferative and apoptotic effect and also decreased tumor growth in xenografted mice	[79]
SSA RC-160	Chinese Hamster Ovary (CHO) DG-44 cells	A Gi/o protein-coupled receptor inhibits cell proliferation via ERK signaling	Inhibition of cell proliferation	[87]
Octreotide	Human HepG2, SMMC-7721, and L-02 cells	Some mechanism most probably associated with SST3	Antiproliferative effect proportional to the SSA dose established	[44]
Receptor agonists	Human HepG2, HuH7, and hepatic stellate cells (HSCs)	Signaling pathways linked to SSTs	Reduced migration of cancer cells but no antiproliferative effect observed	[52]
Octreotide±lamivudine	Human HepG2 and HepG2x	Signaling pathways linked to SSTR2 and SSTR5	Increased apoptotic effect on the HepG2 cell line	[82]
L-779,976	HEK 293 cell clone	Inhibiting adenylate cyclase, activating ERK1/2, and inducing the cyclin-dependent kinase inhibitor p27(Kip1)	Inducement of cell cycle arrest	[88]
Octreotide	SMMC-7221 cells	Activation of the Fas-FasL ligand system	Inducement of apoptosis	[47]
Octreotide	Human Bel-7402	Some mechanisms linked to SSTR2	No antiproliferative effect but no xenografted HCC development	[83]
Octreotide	Human HepG2 cells	Caspase-mediated signaling pathways	Inhibition of proliferation at high octreotide doses and proliferation of promotion at low octreotide doses	[96]
Octreotide	Rat hepatic stellate cells (HSCs)	A mechanism related to the cytokine environment of HSCs	Varied effect. General reduction in collagen synthesis related to PDFG and TGFb1	[85]
Octreotide, lanreotide, SOM230	Human Bel-7402 cells	Some mechanisms linked to SSTR expression variation	Apoptotic effect observed and improved survivability and life quality after the xenograft on mice	[84]

**Table 4:** Clinical trials evaluating the role of SSAs in the treatment of HCC.

Somatostatin analog	Trial type	Trial length	Patients/controls	Result	References
Octreotide	R	≤4 years	28/30	[P] Median survival levels of treated patients increased significantly	[77]
Lanreotide	NR	Variable	21/0	[N] Insignificant improvement in most patients; minor life quality improvement of some patients	[78]
Octreotide	R	Variable	12/13	[P] Overall increase in the survivability of treated patients	[129]
Octreotide	R	7 mo.	35/35	[N] No tumor regression, and no improvement in life quality of the patients	[137]
Octreotide/lanreotide	NR	n/a	32/27	[P] Overall improved survival rate of the SSA-treated patients and superior life quality	[130]
Octreotide	NR	6 mo.	63/0	[N] No significant prolongation of survival observed	[156]
Octreotide+tamoxifen	R	3 mo.	24/15	[P] Response of 43% of the patients treated with octreotide and doubling of their survival	[157]
Octreotide	R	Variable	32/33	[P] Improvement of the survival rate of the treated group	[131]
Octreotide	NR	Variable	30/0	[P] Increase of survivability and life quality of patients	[136]
Octreotide	R	Variable	20/25	[P] Improvement of the survival rate of the treated group	[132]
Octreotide	NR	32 mo.	41/33	[N] Similar survivability between the treated patients and the control group treated with TACE	[140]
Octreotide	NR	2 years	26/0	[N] Very limited beneficial response to treatment	[142]
Octreotide	NR	≤12 mo.	63/0	[N] No improvement of patient life quality and minor anticancer activity of octreotide	[144]
Octreotide±rofecoxib	R	min. 6 mo.	71/0	[P] Increased survivability in patients with high IGF and VEGF levels	[149]
Octreotide	R	3 years	31/30	[P] Response of those patients expressing SSTRs and doubling of the survival rate	[145]
Octreotide+tamoxifen	R	Variable	56/53	[N] No clear benefits in patient survival	[147]
Octreotide	R	Variable	60/59	[N] No significant improvement and no objective tumor regression	[138]
Octreotide	R	Variable	16/14	[P] Moderate increase of the survival rate of the treated group	[134]
Octreotide	NR	54 mo.	35/0	[P] Significant tumor regression (14%) and clear clinical benefits (80%) in association with VEGF levels	[154]
Octreotide	NR	72 mo.	95/0	[P] Positive results for the group receiving the octreotide treatment	[127]
Octreotide	NR	30 mo.	22/0	[P] Positive results for 6 patients of Asian descent who had hepatitis B-induced cirrhosis	[146]
Octreotide	R	2 years	135/137	[N] No improvement in patient survival rate and negative consequence on patient life quality	[139]
Octreotide+sorafenib	NR	Variable	50/0	[P] Slightly positive results on the survivability of some patients	[148]
Octreotide	R	Variable	21/24	[P] Increase of survival rate of the treated group and significant 1-year survival increase	[133]
Octreotide+sorafenib	NR	Variable	50/0	[P] Reduction of oxidative stress in the treated group, potentially signifying an antineoplastic effect	[153]
Octreotide+heparin	NR	1 year	84/63	[P] Significant reduction in tumor metastasis of the treated group	[150]
Octreotide	NR	5 years	99/0	[P] Higher survivability in patients with higher SSTR expression	[155]
Pasireotide+everolimus	NR	Variable	24/0	[N] No clear benefit from the combination of pasireotide and everolimus was discerned	[151]
Octreotide+celecoxib	R	3 years	35/36	[P] Prolonged overall survival, enhanced tumor response, and reduced postembolization syndrome of the treated patients	[152]
Pasireotide	NR	≤54 mo.	20/0	[N] Limited clinical benefit of pasireotide as a second- or third-line treatment	[141]

Abbreviations: [P]: positive results; [N]: negative results; R: randomized study; NR: nonrandomized study; mo.: months; n/a: not available.

## Data Availability

No data were used to support this study.

## References

[1] Reynaert H., Colle I. (2019). Treatment of advanced hepatocellular carcinoma with somatostatin analogues: a review of the literature. *International journal of molecular sciences*.

[2] Midorikawa Y., Takayama T., Higaki T. (2016). Early hepatocellular carcinoma as a signaling lesion for subsequent malignancy. *Japanese journal of clinical oncology*.

[3] Midorikawa Y., Takayama T., Shimada K. (2013). Marginal survival benefit in the treatment of early hepatocellular carcinoma. *Journal of Hepatology*.

[4] Ebisawa K., Midorikawa Y., Higaki T. (2016). Natural history of nonenhancing lesions incidentally detected during the diagnosis of hepatocellular carcinoma. *Surgery*.

[5] Yagi R., Midorikawa Y., Moriguchi M. (2018). Liver resection for recurrent hepatocellular carcinoma to improve survivability: a proposal of indication criteria. *Surgery*.

[6] Balogh J., Victor D., Asham E. H. (2016). Hepatocellular carcinoma: a review. *Journal of hepatocellular carcinoma*.

[7] Batey R. G., Burns T., Benson R. J., Byth K. (1992). Alcohol consumption and the risk of cirrhosis. *The Medical Journal of Australia*.

[8] Morgan T. R., Mandayam S., Jamal M. M. (2004). Alcohol and hepatocellular carcinoma. *Gastroenterology*.

[9] Choe J. W., Hyun J. J., Kim B., Han K. D. (2021). Influence of metabolic syndrome on cancer risk in HBV carriers: a nationwide population based study using the National Health Insurance Service database. *Journal of clinical medicine*.

[10] Jeon H., Kim J. H., Lee S. S. (2021). Impact of acute kidney injury on survival in patients with chronic hepatitis C: a retrospective cohort study. *BMC Infectious Diseases*.

[11] Wiese S., Voiosu A., Hove J. D. (2020). Fibrogenesis and inflammation contribute to the pathogenesis of cirrhotic cardiomyopathy. *Alimentary Pharmacology & Therapeutics*.

[12] Scheau A. E., Scheau C., Lupescu I. G. (2017). Nodule-in-nodule imaging pattern in hepatocellular carcinoma treated by transarterial chemoembolization - a multiparametric magnetic resonance imaging study. *Journal of Gastrointestinal & Liver Diseases*.

[13] Okuno M., Newhook T. E., Joechle K. (2020). Characteristics of atypical large well-differentiated hepatocellular carcinoma: a specific subtype of hepatocellular carcinoma?. *HPB*.

[14] Lin S., Hoffmann K., Schemmer P. (2012). Treatment of hepatocellular carcinoma: a systematic review. *Liver Cancer*.

[15] El-Serag H. B., Marrero J. A., Rudolph L., Reddy K. R. (2008). Diagnosis and treatment of hepatocellular carcinoma. *Gastroenterology*.

[16] Davila J. A., Duan Z., McGlynn K. A., El-Serag H. B. (2012). Utilization and outcomes of palliative therapy for hepatocellular carcinoma: a population-based study in the United States. *Journal of Clinical Gastroenterology*.

[17] Clark H. P., Carson W. F., Kavanagh P. V., Ho C. P. H., Shen P., Zagoria R. J. (2005). Staging and current treatment of hepatocellular carcinoma. *Radiographics*.

[18] Cabrera R., Nelson D. R. (2010). Review article: the management of hepatocellular carcinoma. *Alimentary Pharmacology & Therapeutics*.

[19] El-Serag H. B. (2011). Hepatocellular carcinoma. *The New England Journal of Medicine*.

[20] Bruix J., Sherman M. (2005). Management of hepatocellular carcinoma. *Hepatology*.

[21] Escudier B., Worden F., Kudo M. (2019). Sorafenib: key lessons from over 10 years of experience. *Expert Review of Anticancer Therapy*.

[22] Liu Z., Lin Y., Zhang J. (2019). Molecular targeted and immune checkpoint therapy for advanced hepatocellular carcinoma. *Journal of Experimental & Clinical Cancer Research*.

[23] Chen Z., Xie H., Hu M. (2020). Recent progress in treatment of hepatocellular carcinoma. *American Journal of Cancer Research*.

[24] Popescu G. D., Scheau C., Badarau I. A. (2021). The effects of capsaicin on gastrointestinal cancers. *Molecules*.

[25] Scheau C., Badarau I. A., Caruntu C. (2019). Capsaicin: effects on the pathogenesis of hepatocellular carcinoma. *Molecules*.

[26] Hu Y., Wang S., Wu X. (2013). Chinese herbal medicine-derived compounds for cancer therapy: a focus on hepatocellular carcinoma. *Journal of Ethnopharmacology*.

[27] Scheau C., Mihai L. G., Bădărău I. A., Caruntu C. (2020). Emerging applications of some important natural compounds in the field of oncology. *Farmácia*.

[28] Bloom S. R., Polak J. M. (1987). Somatostatin. *BMJ*.

[29] Cervia D., Casini G., Bagnoli P. (2008). Physiology and pathology of somatostatin in the mammalian retina: a current view. *Molecular and Cellular Endocrinology*.

[30] Scheau C., Draghici C., Ilie M. A. (2021). Neuroendocrine factors in melanoma pathogenesis. *Cancers*.

[31] Rai U., Thrimawithana T. R., Valery C., Young S. A. (2015). Therapeutic uses of somatostatin and its analogues: current view and potential applications. *Pharmacology & Therapeutics*.

[32] Bloom S. R., Mortimer C. H., Thorner M. O. (1974). Inhibition of gastrin and gastric-acid secretion by growth-hormone release- inhibiting hormone. *The Lancet*.

[33] Arimura A., Sato H., Dupont A., Nishi N., Schally A. V. (1975). Somatostatin: abundance of immunoreactive hormone in rat stomach and pancreas. *Science*.

[34] Keast J. R., Furness J. B., Costa M. (1984). Somatostatin in human enteric nerves. *Cell and Tissue Research*.

[35] Polak J. M., Bloom S. R. (1986). Somatostatin localization in tissues. *Scandinavian Journal of Gastroenterology. Supplement*.

[36] Patel Y. C., Reichlin S. (1978). Somatostatin in hypothalamus, extrahypothalamic brain, and peripheral tissues of the rat. *Endocrinology*.

[37] Jiang S. H., Zhang X. X., Hu L. P. (2020). Systemic regulation of cancer development by neuro-endocrine-immune signaling network at multiple levels. *Frontiers in Cell and Developmental Biology*.

[38] Mancini S., Alboni S., Mattei G. (2020). Preliminary results of a multidisciplinary Italian study adopting a psycho-neuro-endocrine-immunological (PNEI) approach to the study of colorectal adenomas. *Acta Bio-Medica*.

[39] Dasgupta P. (2004). Somatostatin analogues: multiple roles in cellular proliferation, neoplasia, and angiogenesis. *Pharmacology & Therapeutics*.

[40] Bousquet C., Guillermet J., Vernejoul F., Lahlou H., Buscail L., Susini C. (2004). Somatostatin receptors and regulation of cell proliferation. *Digestive and Liver Disease*.

[41] Ferjoux G., Bousquet C., Cordelier P. (2000). Signal transduction of somatostatin receptors negatively controlling cell proliferation. *Journal of Physiology, Paris*.

[42] War S. A., Kumar U. (2012). Coexpression of human somatostatin receptor-2 (SSTR2) and SSTR3 modulates antiproliferative signaling and apoptosis. *Journal of Molecular Signaling*.

[43] Kouroumalis E., Samonakis D., Notas G. (2018). Somatostatin in hepatocellular carcinoma: experimental and therapeutic implications. *Hepatoma Research*.

[44] Liu H. L., Huo L., Wang L. (2004). Octreotide inhibits proliferation and induces apoptosis of hepatocellular carcinoma cells. *Acta Pharmacologica Sinica*.

[45] Lasfer M., Vadrot N., Schally A. V. (2005). Potent induction of apoptosis in human hepatoma cell lines by targeted cytotoxic somatostatin analogue AN-238. *Journal of Hepatology*.

[46] Teijeiro R., Rios R., Costoya J. A. (2002). Activation of human somatostatin receptor 2 promotes apoptosis through a mechanism that is independent from induction of p53. *Cellular Physiology and Biochemistry*.

[47] Ma Q., Meng L. Q., Liu J. C. (2008). Octreotide induces apoptosis of human hepatoma cells by the mechanism of facilitating the Fas/FasL gene expression therein. *Zhonghua Yi Xue Za Zhi*.

[48] Dalm V. A., Hofland L. J., Lamberts S. W. (2008). Future clinical prospects in somatostatin/cortistatin/somatostatin receptor field. *Molecular and Cellular Endocrinology*.

[49] Pintér E., Helyes Z., Szolcsányi J. (2006). Inhibitory effect of somatostatin on inflammation and nociception. *Pharmacology & Therapeutics*.

[50] Chao T. C., Chao H. H., Chen M. F., Lin J. D. (1997). Somatostatin modulates the function of Kupffer cells. *Regulatory Peptides*.

[51] Lang A., Sakhnini E., Fidder H. H., Maor Y., Bar-Meir S., Chowers Y. (2005). Somatostatin inhibits pro-inflammatory cytokine secretion from rat hepatic stellate cells. *Liver International*.

[52] Reynaert H., Rombouts K., Vandermonde A. (2004). Expression of somatostatin receptors in normal and cirrhotic human liver and in hepatocellular carcinoma. *Gut*.

[53] Reubi J. C., Waser B., Cescato R., Gloor B., Stettler C., Christ E. (2010). Internalized somatostatin receptor subtype 2 in neuroendocrine tumors of octreotide-treated patients. *The Journal of Clinical Endocrinology and Metabolism*.

[54] Ye Y., Huang A., Huang C. (2013). Comparative mitochondrial proteomic analysis of hepatocellular carcinoma from patients. *Proteomics. Clinical Applications*.

[55] Lamiman K., Keller J. M., Mizokami A., Zhang J., Keller E. T. (2014). Survey of Raf kinase inhibitor protein (RKIP) in multiple cancer types. *Critical Reviews in Oncogenesis*.

[56] Huang C. Z., Huang A. M., Liu J. F., Wang B., Lin K. C., Ye Y. B. (2018). Somatostatin octapeptide inhibits cell invasion and metastasis in hepatocellular carcinoma through PEBP1. *Cellular Physiology and Biochemistry*.

[57] Fingleton B. (2008). MMPs as therapeutic targets—still a viable option?. *Seminars in Cell & Developmental Biology*.

[58] Corbley M. J., Frank D. A. (2012). Protein therapeutics in oncology. *Signaling Pathways in Cancer Pathogenesis and Therapy*.

[59] Amălinei C., Căruntu I. D., Giuşcă S. E., Bălan R. A. (2010). Matrix metalloproteinases involvement in pathologic conditions. *Romanian Journal of Morphology and Embryology*.

[60] di Nezza L. A., Misajon A., Zhang J. (2002). Presence of active gelatinases in endometrial carcinoma and correlation of matrix metalloproteinase expression with increasing tumor grade and invasion. *Cancer*.

[61] Scheau C., Badarau I. A., Costache R. (2019). The role of matrix metalloproteinases in the epithelial-mesenchymal transition of hepatocellular carcinoma. *Analytical Cellular Pathology*.

[62] Devy L., Huang L., Naa L. (2009). Selective inhibition of matrix metalloproteinase-14 blocks tumor growth, invasion, and angiogenesis. *Cancer Research*.

[63] Sevket O., Sevket A., Molla T. (2013). Somatostatin analogs regress endometriotic implants in rats by decreasing implant levels of vascular endothelial growth factor and matrix metaloproteinase 9. *Reproductive Sciences*.

[64] Raderer M., Hejna M. H., Kurtaran A. (1999). Successful treatment of an advanced hepatocellular carcinoma with the long-acting somatostatin analog lanreotide. *The American Journal of Gastroenterology*.

[65] Borbath I., Lhommel R., Guiot Y., Coche E., Sempoux C. (2012). Lanreotide treatment of metastatic hepatocellular carcinoma resulting in partial regression and more than 3 years of progression-free survival. *Acta Gastroenterologica Belgica*.

[66] Lamberts S. W., van der Lely A. J., de Herder W. W., Hofland L. J. (1996). Octreotide. *The New England Journal of Medicine*.

[67] Guillemin R. (1978). Control of adenohypophysial functions by peptides of the central nervous system. *Harvey Lectures*.

[68] Lamberts S. W., Uitterlinden P., Verschoor L., van Dongen K. J., del Pozo E. (1985). Long-term treatment of acromegaly with the somatostatin analogue SMS 201-995. *The New England Journal of Medicine*.

[69] Binmoeller K. F., Harris A. G., Dumas R., Grimaldi C., Delmont J. P. (1992). Does the somatostatin analogue octreotide protect against ERCP induced pancreatitis?. *Gut*.

[70] Reynaert H., Geerts A. (2003). Pharmacological rationale for the use of somatostatin and analogues in portal hypertension. *Alimentary Pharmacology & Therapeutics*.

[71] Schmid H. A., Schoeffter P. (2004). Functional activity of the multiligand analog SOM230 at human recombinant somatostatin receptor subtypes supports its usefulness in neuroendocrine tumors. *Neuroendocrinology*.

[72] Lequoy M., Desbois-Mouthon C., Wendum D. (2017). Somatostatin receptors in resected hepatocellular carcinoma: status and correlation with markers of poor prognosis. *Histopathology*.

[73] Verhoef C., van Dekken H., Hofland L. J. (2008). Somatostatin receptor in human hepatocellular carcinomas: biological, patient and tumor characteristics. *Digestive Surgery*.

[74] Bläker M., Schmitz M., Gocht A. (2004). Differential expression of somatostatin receptor subtypes in hepatocellular carcinomas. *Journal of Hepatology*.

[75] Kaemmerer D., Schindler R., Mußbach F. (2017). Somatostatin and CXCR4 chemokine receptor expression in hepatocellular and cholangiocellular carcinomas: tumor capillaries as promising targets. *BMC Cancer*.

[76] Chou C. K., Ho L. T., Ting L. P. (1987). Selective suppression of insulin-induced proliferation of cultured human hepatoma cells by somatostatin. *The Journal of Clinical Investigation*.

[77] Kouroumalis E., Skordilis P., Thermos K., Vasilaki A., Moschandrea J., Manousos O. N. (1998). Treatment of hepatocellular carcinoma with octreotide: a randomised controlled study. *Gut*.

[78] Raderer M., Hejna M. H., Muller C. (2000). Treatment of hepatocellular cancer with the long acting somatostatin analog lanreotide in vitro and in vivo. *International Journal of Oncology*.

[79] Wang C., Tang C., Tang L. (2001). Inhibition effects of octreotide on the growth of hepatocellular carcinoma in vitro and in vivo. *Zhonghua Yi Xue Za Zhi*.

[80] Chen X., Liu Z., Ai Z. (2001). Antineoplastic mechanism of octreotide action in human hepatoma. *Chinese Medical Journal*.

[81] Pan Q., Li D. G., Lu H. M., Lu L. Y., Wang Y. Q., Xu Q. F. (2004). Antiproliferative and proapoptotic effects of somatostatin on activated hepatic stellate cells. *World Journal of Gastroenterology*.

[82] Xie Y., Tang C. W., Wang C. H. (2005). Effect of HBV X gene transfection on octreotide-inhibited growth of hepatocellular carcinoma cell line HepG2. *Ai Zheng*.

[83] Hua Y. P., Yin X. Y., Peng B. G. (2009). Mechanisms and influence of octreotide-induced regulation of somatostatin receptor 2 on hepatocellular carcinoma. *Chemotherapy*.

[84] Lü X. H., Wang C. H., Xie Y. (2017). Differences of therapeutic efficacy between different kinds of somatostatin analogue for primary hepatocellular carcinoma. *Sichuan Da Xue Xue Bao. Yi Xue Ban*.

[85] Klironomos S., Notas G., Sfakianaki O., Kiagiadaki F., Xidakis C., Kouroumalis E. (2014). Octreotide modulates the effects on fibrosis of TNF-*α*, TGF-*β* and PDGF in activated rat hepatic stellate cells. *Regulatory Peptides*.

[86] Florio T., Yao H., Carey K. D., Dillon T. J., Stork P. J. (1999). Somatostatin activation of mitogen-activated protein kinase via somatostatin receptor 1 (SSTR1). *Molecular Endocrinology*.

[87] Lahlou H., Saint-Laurent N., Estève J. P. (2003). sst2 Somatostatin Receptor Inhibits Cell Proliferation through Ras-, Rap1-, and B-Raf-dependent ERK2 Activation∗. *The Journal of Biological Chemistry*.

[88] Grant M., Alturaihi H., Jaquet P., Collier B., Kumar U. (2008). Cell growth inhibition and functioning of human somatostatin receptor type 2 are modulated by receptor heterodimerization. *Molecular Endocrinology*.

[89] Patel Y. C. (1999). Somatostatin and its receptor family. *Frontiers in Neuroendocrinology*.

[90] BRUNS C., WECKBECKER G., RAULF F. (1994). Molecular pharmacology of somatostatin-receptor subtypes. *Annals of the New York Academy of Sciences*.

[91] Florio T., Scorziello A., Fattore M. (1996). Somatostatin inhibits PC Cl3 thyroid cell proliferation through the modulation of phosphotyrosine phosphatase activity:. *The Journal of Biological Chemistry*.

[92] Møller L. N., Stidsen C. E., Hartmann B., Holst J. J. (2003). Somatostatin receptors. *Biochimica et Biophysica Acta*.

[93] Cordelier P., Esteve J. P., Bousquet C. (1997). Characterization of the antiproliferative signal mediated by the somatostatin receptor subtype sst5. *PNAS*.

[94] Theodoropoulou M., Zhang J., Laupheimer S. (2006). Octreotide, a somatostatin analogue, mediates its antiproliferative action in pituitary tumor cells by altering phosphatidylinositol 3-kinase signaling and inducing Zac1 expression. *Cancer Research*.

[95] Neel B. G., Tonks N. K. (1997). Protein tyrosine phosphatases in signal transduction. *Current Opinion in Cell Biology*.

[96] Tsagarakis N. J., Drygiannakis I., Batistakis A. G., Kolios G., Kouroumalis E. A. (2011). Octreotide induces caspase activation and apoptosis in human hepatoma HepG2 cells. *World Journal of Gastroenterology*.

[97] Tong H., Li X., Zhang C. L. (2013). Transcatheter arterial embolization followed by octreotide and celecoxib synergistically prolongs survival of rabbits with hepatic VX2 allografts. *Journal of Digestive Diseases*.

[98] Xie Y., Chen S., Wang C. H., Tang C. W. (2011). SOM230 combined with celecoxib prolongs the survival in nude mice with HepG-2 xenografts. *Cancer Biology & Therapy*.

[99] Volpe E., Sambucci M., Battistini L., Borsellino G. (2016). Fas-Fas ligand: checkpoint of T cell functions in multiple sclerosis. *Frontiers in Immunology*.

[100] Nagata S. (1997). Apoptosis by death factor. *Cell*.

[101] Nagata S., Golstein P. (1995). The Fas death factor. *Science*.

[102] Wolf B. B., Green D. R. (1999). Suicidal tendencies: apoptotic cell death by caspase family proteinases. *The Journal of Biological Chemistry*.

[103] Notas G., Kampa M., Nifli A. P. (2007). The inhibitory effect of opioids on HepG2 cells is mediated via interaction with somatostatin receptors. *European Journal of Pharmacology*.

[104] Avella D. M., Kimchi E. T., Donahue R. N. (2010). The opioid growth factor-opioid growth factor receptor axis regulates cell proliferation of human hepatocellular cancer. *American Journal of Physiology. Regulatory, Integrative and Comparative Physiology*.

[105] Adams R. L., Adams I. P., Lindow S. W., Zhong W., Atkin S. L. (2005). Somatostatin receptors 2 and 5 are preferentially expressed in proliferating endothelium. *British Journal of Cancer*.

[106] Jia W. D., Xu G. L., Sun H. C., Wang L., Xu R. N., Xue Q. (2003). Effect of octreotide on angiogenesis induced by hepatocellular carcinoma in vivo. *Hepatobiliary & Pancreatic Diseases International*.

[107] Jia W. D., Xu G. L., Xu R. N. (2003). Octreotide acts as an antitumor angiogenesis compound and suppresses tumor growth in nude mice bearing human hepatocellular carcinoma xenografts. *Journal of Cancer Research and Clinical Oncology*.

[108] Garcia de la Torre N., Wass J. A., Turner H. E. (2002). Antiangiogenic effects of somatostatin analogues. *Clinical Endocrinology*.

[109] Ristori C., Ferretti M. E., Pavan B. (2008). Adenylyl cyclase/cAMP system involvement in the antiangiogenic effect of somatostatin in the retina. Results from transgenic mice. *Neurochemical Research*.

[110] Gao J. H., Wen S. L., Feng S. (2016). Celecoxib and octreotide synergistically ameliorate portal hypertension via inhibition of angiogenesis in cirrhotic rats. *Angiogenesis*.

[111] Lamberts S. W., de Herder W. W., Hofland L. J. (2002). Somatostatin analogs in the diagnosis and treatment of cancer. *Trends in Endocrinology and Metabolism*.

[112] Lattuada D., Casnici C., Crotta K. (2007). Inhibitory effect of pasireotide and octreotide on lymphocyte activation. *Journal of Neuroimmunology*.

[113] Valatas V., Kolios G., Manousou P. (2004). Secretion of inflammatory mediators by isolated rat Kupffer cells: the effect of octreotide. *Regulatory Peptides*.

[114] Xidakis C., Kolios G., Valatas V., Notas G., Mouzas I., Kouroumalis E. (2004). Effect of octreotide on apoptosis-related proteins in rat Kupffer cells: a possible anti-tumour mechanism. *Anticancer Research*.

[115] Abdel-Hamid N. M., Mohafez O. M., Nazmy M. H., Farhan A., Thabet K. (2015). The effect of co-administration of Lawsonia inermis extract and octreotide on experimental hepatocellular carcinoma. *Environmental Health and Preventive Medicine*.

[116] Bouchard M. J., Schneider R. J. (2004). The enigmatic X gene of hepatitis B virus. *Journal of Virology*.

[117] Rocheville M., Lange D. C., Kumar U., Sasi R., Patel R. C., Patel Y. C. (2000). Subtypes of the Somatostatin Receptor Assemble as Functional Homo- and Heterodimers∗. *The Journal of Biological Chemistry*.

[118] Reynaert H., Rombouts K., Jia Y. (2005). Somatostatin at nanomolar concentration reduces collagen I and III synthesis by, but not proliferation of activated rat hepatic stellate cells. *British Journal of Pharmacology*.

[119] Schindel D. T., Grosfeld J. L. (1997). Hepatic resection enhances growth of residual intrahepatic and subcutaneous hepatoma, which is inhibited by octreotide. *Journal of pediatric surgery*.

[120] Hua Y. P., Huang J. F., Liang L. J., Li S. Q., Lai J. M., Liang H. Z. (2005). The study of inhibition effect of octreotide on the growth of hepatocellular carcinoma xenografts in situ in nude mice. *Zhonghua Wai Ke Za Zhi*.

[121] Jia W. D., Xu G. L., Wang W. (2009). A somatostatin analogue, octreotide, inhibits the occurrence of second primary tumors and lung metastasis after resection of hepatocellular carcinoma in mice. *The Tohoku Journal of Experimental Medicine*.

[122] Tong H., Li X., Zhang C. L. (2013). Octreotide and celecoxib synergistically encapsulate VX2 hepatic allografts following transcatheter arterial embolisation. *Experimental and Therapeutic Medicine*.

[123] Wang X. X., Ye T., Li M. (2017). Effects of octreotide on hepatic glycogenesis in rats with high fat dietinduced obesity. *Molecular Medicine Reports*.

[124] Borbath I., Leclercq I. A., Abarca-Quinones J. (2007). Inhibition of early preneoplastic events in the rat liver by the somatostatin analog lanreotide. *Cancer Science*.

[125] Borbath I., Leclercq I. A., Sempoux C., Abarca-Quinones J., Desaeger C., Horsmans Y. (2010). Efficacy of lanreotide in preventing the occurrence of chemically induced hepatocellular carcinoma in rats. *Chemico-Biological Interactions*.

[126] Borbath I., Stärkel P. (2011). Chemoprevention of hepatocellular carcinoma. Proof of concept in animal models. *Acta Gastroenterologica Belgica*.

[127] Schöniger-Hekele M., Kettenbach J., Peck-Radosavljevic M., Müller C. (2009). Octreotide treatment of patients with hepatocellular carcinoma - a retrospective single centre controlled study. *Journal of Experimental & Clinical Cancer Research*.

[128] Siveke J. T., Herberhold C., Folwaczny C. (2003). Complete regression of advanced HCC with long acting octreotide. *Gut*.

[129] Wu P., Gu X. Y., Jiang Z. (2001). Efficacy of octreotide in advanced hepatocellular carcinoma: a clinical trial. *Chinese Journal of Hepatobiliary Surgery*.

[130] Samonakis D. N., Moschandreas J., Arnaoutis T. (2002). Treatment of hepatocellular carcinoma with long acting somatostatin analogues. *Oncology Reports*.

[131] Yang M. N., Xiao B., Wang X. L., Xue Y. P. (2003). Effects of octreotide in elderly patients with advanced primary hepatic cancer. *Journal of Jiangsu Clinical Medicine*.

[132] Zhang L., Jiang Z., Li S. Y. (2004). Clinical study of octreodide for advanced primary liver cancer. *Chinese Clinical Oncology*.

[133] Zhang B., Xu F. (2010). The clinical observation of octreotide in the treatment of 45 patients with advanced primary liver carcinoma. *Journal of Basic and Clinical Oncology*.

[134] Ou S. Q., Chen Z. Q., Ma Y. L. (2007). Clinical study of octreotide for advanced hepatocellular carcinoma. *Hainan Medizinhistorisches Journal*.

[135] Kouroumalis E., Samonakis D., Sordilis P. (2003). Octreotide treatment of hepatocellular carcinoma. *Hepatology*.

[136] Patsanas T., Kapetanos D., Ilias A. (2004). Octreotide in the treatment of inoperable hepatocellular carcinoma. *Annals of Gastroenterology*.

[137] Yuen M. F., Poon R. T., Lai C. L. (2002). A randomized placebo-controlled study of long-acting octreotide for the treatment of advanced hepatocellular carcinoma. *Hepatology*.

[138] Becker G., Allgaier H. P., Olschewski M., Zähringer A., Blum H. E., HECTOR Study Group (2007). Long-acting octreotide versus placebo for treatment of advanced HCC: a randomized controlled double-blind study. *Hepatology*.

[139] Barbare J. C., Bouché O., Bonnetain F. (2009). Treatment of advanced hepatocellular carcinoma with long-acting octreotide: a phase III multicentre, randomised, double blind placebo-controlled study. *European Journal of Cancer*.

[140] PLENTZ R. R., TILLMANN H. L., KUBICKA S. (2005). Hepatocellular carcinoma and octreotide: treatment results in prospectively assigned patients with advanced tumor and cirrhosis stage. *Journal of Gastroenterology and Hepatology*.

[141] Feun L. G., Wangpaichitr M., Li Y. Y. (2018). Phase II trial of SOM230 (pasireotide LAR) in patients with unresectable hepatocellular carcinoma. *Journal of hepatocellular carcinoma*.

[142] Slijkhuis W. A., Stadheim L., Hassoun Z. M. (2005). Octreotide therapy for advanced hepatocellular carcinoma. *Journal of Clinical Gastroenterology*.

[143] Samonakis D. N., Christodoulakis N., Kouroumalis E. A. (2006). Octreotide for unresectable hepatocellular carcinoma: beyond the first sight. *Journal of Clinical Gastroenterology*.

[144] Cebon J., Findlay M., Hargreaves C. (2006). Somatostatin receptor expression, tumour response, and quality of life in patients with advanced hepatocellular carcinoma treated with long-acting octreotide. *British Journal of Cancer*.

[145] Dimitroulopoulos D., Xinopoulos D., Tsamakidis K. (2007). Long acting octreotide in the treatment of advanced hepatocellular cancer and overexpression of somatostatin receptors: randomized placebo-controlled trial. *World Journal of Gastroenterology*.

[146] Shah U., O’Neil B., Allen J. (2009). A phase II study of long-acting octreotide in patients with advanced hepatocellular carcinoma and CLIP score of 3 or higher. *Gastrointestinal cancer research*.

[147] Verset G., Verslype C., Reynaert H. (2007). Efficacy of the combination of long-acting release octreotide and tamoxifen in patients with advanced hepatocellular carcinoma: a randomised multicentre phase III study. *British Journal of Cancer*.

[148] Prete S. D., Montella L., Caraglia M. (2010). Sorafenib plus octreotide is an effective and safe treatment in advanced hepatocellular carcinoma: multicenter phase II So.LAR. study. *Cancer Chemotherapy and Pharmacology*.

[149] Treiber G., Wex T., Röcken C., Fostitsch P., Malfertheiner P. (2006). Impact of biomarkers on disease survival and progression in patients treated with octreotide for advanced hepatocellular carcinoma. *Journal of Cancer Research and Clinical Oncology*.

[150] Jia W., Feng K., Fan P. (2012). Post-TACE combination therapy of heparin and octreotide results in decreased tumor metastasis in extrahepatic tumorigenesis. *Cell Biochemistry and Biophysics*.

[151] Sanoff H. K., Kim R., Ivanova A., Alistar A., McRee A. J., O’Neil B. H. (2015). Everolimus and pasireotide for advanced and metastatic hepatocellular carcinoma. *Investigational New Drugs*.

[152] Tong H., Wei B., Chen S. (2017). Adjuvant celecoxib and lanreotide following transarterial chemoembolisation for unresectable hepatocellular carcinoma: a randomized pilot study. *Oncotarget*.

[153] Caraglia M., Giuberti G., Marra M. (2011). Oxidative stress and ERK1/2 phosphorylation as predictors of outcome in hepatocellular carcinoma patients treated with sorafenib plus octreotide LAR. *Cell Death & Disease*.

[154] Montella L., Addeo R., Caraglia M. (2008). Vascular endothelial growth factor monitoring in advanced hepatocellular carcinoma patients treated with radiofrequency ablation plus octreotide: a single center experience. *Oncology Reports*.

[155] Liu Y., Jiang L., Mu Y. (2013). Somatostatin receptor subtypes 2 and 5 are associated with better survival in operable hepatitis B-related hepatocellular carcinoma following octreotide long-acting release treatment. *Oncology Letters*.

[156] Rabe C., Pilz T., Allgaier H. P. (2002). Clinical outcome of a cohort of 63 patients with hepatocellular carcinoma treated with octreotide. *Zeitschrift für Gastroenterologie*.

[157] Pan D. Y., Qiao J. G., Chen J. W., Huo Y. C., Zhou Y. K., Shi H. A. (2003). Tamoxifen combined with octreotide or regular chemotherapeutic agents in treatment of primary liver cancer: a randomized controlled trial. *Hepatobiliary & Pancreatic Diseases International*.

